# Supplementary material for: Are carbon emissions trading and green financial instruments synergistic? -Comprehensive quantitative research based on content analysis
Source: PLoS One. 2024 Mar 7;19(3):e0298601. doi: 10.1371/journal.pone.0298601 (PMC10919601; doi:10.1371/journal.pone.0298601)
Supplement: S1 Data — (ZIP) [file pone.0298601.s001.zip › Data/地级市Z_F工作报告.zip!/╔╜╬≈/│ñ╓╬╩╨/└┤╫╘╣1⁄2╓┌║┼í╛┬φ┐╦╩2╛▌═°í┐_2014.docx]

各位代表：

现在，我代表市人民政府向大会报告工作，请予审议，并请市政协委员和其他列席人员提出意见。

2013年工作回顾

2013年，在市委的坚强领导下，全市上下紧紧围绕实施“五五战略”、率先全面小康的发展目标，攻坚克难、逆势而上，全力保增长、上项目、抓转型、惠民生、强安全、促稳定，较好地完成了年初确定的各项预期目标任务，经济社会发展取得了来之不易的新成效。

——经济运行稳中向好。全年地区生产总值完成1333.7亿元、增长8.5％，公共财政预算收入完成148.7亿元、增长11.4％，总量分别位居全省第二和第三。居民消费价格涨幅控制在3.2％以内。城镇新增就业5万人，城镇登记失业率1.65％，低于省控目标。全社会用电量达到112.6亿千瓦时、增长2％，实现正增长。公路货运量完成7390.9万吨、增长14.6％，增幅位居全省第二。

——产业转型成效明显。粮食产量超过16亿公斤，再创历史新高。新兴产业完成增加值120亿元，占工业增加值的比重达到14.2%，较上年提高1个百分点。非煤产业占工业增加值的比重达到31%，较上年提高3个百分点。非煤县地区生产总值的比重继续增加，区域发展的协调性不断增强。

——居民收入稳定增长。城镇居民人均可支配收入达到22803元、增长9.9%。农村居民人均纯收入达到9119元、增长12.3%，连续四年快于城镇居民收入增幅。两个收入分别比全省平均水平高347元和1965元。

——社会事业蓬勃发展。先后荣获国家森林城市、国家公共文化服务体系示范区、全国全民健身示范城市、国家智慧城市试点市、全国创建幸福家庭活动试点市和全国法治城市创建活动先进单位等多个国家级荣誉称号。在全省各行业系统考核评比中，我市有35个部门的工作实现了“保三争一”。过去的一年，政府工作主要着力于以下六个方面：

一、确保经济运行处于合理区间

抓传统产业的稳定支撑。及时出台了保障工业运行12条、促进煤炭生产10条和煤电互保等一系列政策措施，组织开展了三个“百日大会战”，推动企业生产提效。加快煤矿技改，全年竣工投产矿井14座，新增产能1335万吨，实现了煤炭产量、增加值、外销量的同步增长。加快焦炭行业重组，整合产能600余万吨，实现了焦炭产能向优势企业集中。加快电力行业“上大压小”，漳泽电厂改扩建、欣隆、高河、赵庄低热值发电和协鑫煤电一体化等新上电厂项目推进顺利。促成大型焦炭、冶金企业与电厂建立了直供电关系。全年煤、焦、冶、电等传统产业完成增加值708亿元、增长10.9%，拉动全市经济增长4.6个百分点。

抓新兴产业的做强做大。重点实施了“千企百强”工程，倾力打造引领经济发展的旗舰团队。紧紧围绕七大新兴产业板块，加快推进了成功汽车、日盛达光伏玻璃、山力铂纳橡胶、康宝生物雪莲、高科LED、达利食品、潞宝金和生、太行山大峡谷等190个新兴产业项目的建设和投产，共完成投资367亿元、增长50.4%，占工业总投资的67.2%。

大力扶持中小微企业发展，免征5400多户小微企业增值税和营业税，新培育销售收入超亿元的“小巨人”企业18户，新创办小微企业2107户，全年民营经济增加值达到537.4亿元、增长18.1%，高于地区生产总值增速9.6个百分点。

抓现代农业的基础夯实。重点实施了“双十”增收富民工程，新增设施蔬菜10.2万亩，占全省新增面积的50%以上。水果、干果经济林和中药材的种植面积大幅增加。农业产业化龙头企业新上项目144个、完成投资53.2亿元，占农业项目总投资的46.5%。新增省级“一村一品”专业村226个、增加53%。绿色食品、无公害农产品、有机食品认证数量名列全省第一。全年农产品加工行业实现增加值28.3亿元、增长17.3%，成为我市继煤炭、焦炭和冶金行业之后的第四大工业行业。

抓服务业的有效拉动。积极开展了各类消费促销活动，加快现代物流、鲜活农产品流通和商业便民体系建设，全社会消费品零售总额完成425.8亿元、增长14.3%。建立了十大出口企业集群，新认定4个省级外贸转型示范基地，进出口总额再次突破10亿美元。全年旅游总收入达到211.7亿元、增长27.2%。全市服务业增加值占地区生产总值的比重达到30.7%，较上年提高2.1个百分点。

抓项目建设的持续带动。组织开展“双月行动”，扎实推进招商引资和项目建设，先后引进了中冶集团高性能取向硅钢、中技金谷低碳建筑示范项目、阿里巴巴·山西长子产业带平台等一批国内一流的高端绿色转型项目。美国AP空气化工公司、德国杜尔集团、西门子公司等知名企业落户我市。全年招商引资实际到位资金725.6亿元、增长23%，项目建设“六位一体”全部超额完成省下达任务，带动全社会固定资产投资完成1086.8亿元、增长25.4%。

二、推进转型综改有实质性进展

推进行政审批制度改革。全年共取消24项、下放29项市级行政审批项目。开展了项目集中审批办结月活动。以房地产项目审批改革为先导，再造审批流程，审批时限由原来400多个缩短至38个工作日，精简审批部门29%，精简审批事项42%，精简中介机构38%，精简办事环节81%。同时，完善了“一站式、保姆式”服务和严格问责制，提高了审批效率，改善了发展环境，规范了政府行为。

推进土地管理制度改革。规范土地一级市场，加大土地收储力度，严格“招拍挂”土地出让程序。开展了违法占地、违法建设专项治理行动，叫停所有违法行为，对首批“两违”项目进行了依法分类处置。出台了关于城区、郊区、高新区统一的城中村改造实施意见，为全面启动、严格规范城中村改造工作奠定了坚实基础。扎实开展了城乡建设用地增减挂钩、矿业存量土地整合利用、工矿废弃地复垦利用等试点工作，为全市拓展用地空间近3万亩。

推进金融体制创新。全市95家企业在山西股权交易中心挂牌，占全省挂牌企业总量的12.5%。中信银行、晋商银行在我市设立分行，长治商业银行成功改制为长治银行，长子、长治县的农信社改制为农村商业银行。襄垣、武乡、长治县组建的3支股权投资基金及基金管理公司，首期募集资金规模达23亿元，为推进城镇化建设提供了有力支持。金融业增加值完成52.8亿元、增长13.6%，缴纳税金7.7亿元、增长85%。

推进产业创新驱动。在全省率先形成“政校企联合、产学研一体”发展模式，与清华大学、天津大学、华南理工大学等161所知名院校和科研院所建立了合作关系，合办科研机构29个；市级以上企业技术研发中心达到49个，博士（院士）工作站达到30家，实施省级以上科技创新项目77项。全年专利申请量1654件、增长21.5%，授权量781件、增长13%，签订各类技术合同76项、合同成交额3.1亿元。

三、加大城市建设和民生改善力度

主城区九大城建重点工程基本完成，构筑起“三环八纵十二横”的路网骨架，道路贯通率达到80%以上。改造和新增燃气管道53公里，13万户市民置换了天然气。新增集中供热面积460万平方米，改造供水管网38.2公里，铺设入地电缆38.9公里，新安装路灯7500余盏、亮化道路37条，新增绿地面积21万平方米。新购置64辆公交车，万人拥有量达到11.54标台，排名全省第一。主城区城建工程完成投资40亿元、拆迁55万平方米，分别相当于过去5年、10年的总和。11个县城开工建设重点城建工程107项，53个重点镇和240个中心村的建设分别完成投资13.1亿元和8.5亿元。全市城镇化率达到46.87%，比上年提高1.56个百分点。

教育惠民工程投资4.6亿元，使近50万名学生受益。通过校企对接，近5000名职业学校毕业生在我市240家企业就业。“好娃娃”工程深入推进，在全国率先实施新生儿出生缺陷干预救助健苗工程，免费为近2万名新生儿进行了血样检测，对971名新生儿开展了干预救助。新开工各类保障性住房20577套，基本建成18030套。全市公益文化设施建设达标率达到87.1%，名列全省第一。

精准实施“百企千村”产业扶贫、移民扶贫、专项扶贫、科教扶贫、定点扶贫五大扶贫开发工程。48家参与百企千村产业扶贫的企业，共实施项目54个，完成投资23.4亿元。12429名山庄窝铺的贫困群众搬出大山，进城入镇，占到全省年度搬迁总人数的12.4%。全市又有4.3万贫困人口稳定脱贫。

四、提升生态建设和环境保护水平

全力推进“六大”林业生态建设工程，完成营造林44万亩，超额完成省定目标任务46.6个百分点。主城区绿化覆盖率达到45.3%，超出省定目标0.5个百分点。实施了保护“母亲河”、“生命泉”工程，浊漳河南源店上段人工湿地工程基本完成，辛安泉文王山地垒河段防污整治工程进展顺利。强力推进全市工业企业的环保达标，实施了162个重点节能改造项目，淘汰落后产能85.3万吨。全市万元地区生产总值能耗下降3.5%，6项主要污染物的减排全部完成省定目标任务。扎实开展了城市“三项治理”和农村环境卫生集中整治。启动了“向污染宣战、享碧水蓝天”大气污染防治百日攻坚行动，今年以来主城区大气综合质量指数同比下降24.5%，主要污染物PM2.5浓度同比下降33.9%。

五、促进安全生产和社会治理不断进步

进一步落实政府和企业“两个主体”责任，扎实开展了安全大检查、专家巡检会诊等活动。按照“四不两直”的要求，在全省率先实行市政府党组及班子成员夜查突查安全生产工作制度。大力推行“手指口述”一线操作法和高危行业现场考试制度，不断夯实全员培训基础。全市各类生产安全事故起数和死亡人数实现了“双下降”，地方煤矿百万吨死亡率和亿元地区生产总值死亡率均低于省控目标。

深入开展“平安长治”建设，积极排查化解社会矛盾，认真做好信访和人民调解工作。健全社会治安防控体系，依法打击各类违法犯罪活动。扎实开展食品药品安全专项整治，切实保障了全市人民饮食用药安全。有效开展“六五”普法，“法治长治”建设取得新成效。积极应对夏季特大洪涝灾害，下拨救灾资金9000多万元，保障了5万多名受灾群众的基本生活和灾后重建。

六、加强政府自身建设

认真贯彻落实中央“八项规定”和国务院“约法三章”以及省市委的各项要求，出台了关于改进政府工作的十六条规定，规范了政府办事程序。开展了超标办公用房、新建楼堂馆所和公务用车专项整治行动。会议减少44%，文件精简35%，全市“三公经费”下降25.3%。在市直109个单位开展了政风行风评议活动，组织16家社会管理和行政执法部门进行了现场直播的电视问政。加大对重点领域、重大投资项目的审计监察力度，增收节支3.9亿元，核减政府投资1.2亿元。出台了长治市依法行政工作要点等规范性文件，办理行政复议案件22件。自觉执行市人大及其常委会的各项决议、决定，支持人民政协履行职能，认真办理人大代表建议和政协委员提案，办复率达100%。

各位代表，过去一年所取得的成绩是省委、省政府正确领导、亲切关怀的结果，是中共长治市委总揽全局、科学决策的结果，是市人大、市政协大力支持、有效监督的结果，是全市人民齐心协力、奋勇拼搏的结果。在此，我代表市人民政府，向全市人民、驻市部队、武警官兵、公安干警，向各民主党派、工商联、无党派人士和各人民团体，向所有关心支持长治发展的社会各界朋友，表示崇高的敬意和衷心的感谢！

一年来我们更加深切地感受到，长治的发展在全省占有很重的分量，省委、省政府对长治实现“四个率先”寄予厚望，老区人民对实施“五五战略”、建设美丽长治充满期待。我们更加清醒地认识到，要实现“率先建成山西的经济强市”，就必须集中精力谋发展、大干苦干抓发展、创优环境快发展、勇于担当促发展，只有科学发展，才能结构优化、规模做大、总量提升，才能富民强市。要实现 “率先走出资源型经济转型的新路”，就必须以煤为基抓循环、新兴产业上高端、坚定不移上项目、全力以赴抓投产，只有新兴产业真正成为新支柱，才是走出了转型发展的新道路。要实现“率先打造全面小康的升级版”，就必须进一步增强扶贫攻坚的紧迫感、环境保护的危机感、安全稳定的责任感、改善民生的使命感，只有全面加速提升发展建设水平，才能成为资源节约型、环境友好型、城乡协调型、社会和谐型的小康升级版。要实现“率先建成四化一体的新型城镇化范式”，就必须坚持科学规划、合理布局、产城融合、突出特色、城乡统筹、协调发展的原则，狠抓基础设施建设，促进服务业发展，只要一张蓝图绘到底，一任接着一任干，持之以恒，久久为功，上党城镇群就一定会以新型城镇化的范式展现在世人面前。

2014年工作安排

2014年是务实推进“五五战略”的关键一年。今年政府工作的总体要求是：高举中国特色社会主义伟大旗帜，以邓小平理论、“三个代表”重要思想、科学发展观为指导，全面贯彻落实党的十八届三中全会精神，坚持稳中求进、改革创新的总基调，以实施“五五战略”为统领，以实现“四个率先”为目标，以保障和改善民生为根本，深入开展党的群众路线教育实践活动，奋力做好稳增长、抓改革、上项目、促转型、惠民生、强安全、保稳定的各项工作，确保完成今年经济社会发展的各项目标任务。

今年全市经济社会发展的主要预期目标是：地区生产总值增长10%左右，全社会固定资产投资增长23.5%，公共财政预算收入增长10%，社会消费品零售总额增长15%，规模以上工业企业增加值增长12%，城镇居民人均可支配收入增长11%，农民人均纯收入增长12%，城镇新增就业岗位4.3万个，城镇登记失业率控制在4.2%以内，居民消费价格涨幅控制在3.5％左右。

约束性指标是：万元地区生产总值综合能耗、二氧化碳排放量分别下降3.5％和3.7％，二氧化硫、化学需氧量、氨氮、氮氧化合物减排完成省下达任务，烟尘、工业粉尘排放量均下降0.5％，万元工业增加值用水量下降4.9%。

各位代表，今年的经济形势更加复杂多变，面对当前宏观经济持续下行、市场需求持续疲软、产业结构矛盾更加突出的严峻局面，要完成全年的目标任务绝非易事，我们必须提振信心、众志成城、真抓实干、只争朝夕地抓好以下六个方面的工作：

一、多措并举促企业增效益

企业是实体经济中物质生产的主体。经济发展最终要靠实体经济的发展，要靠企业发挥效益。当前，促进企业增效益是稳增长的重中之重。

抓企业投产见效。目前，全市356家规模以上工业企业中有35%处于停产半停产状态，还有一部分企业生产经营也很不稳定。同时，按计划今年还有290个企业项目应建成投产。这些企业、项目是支撑长治经济发展的主力军，是决定长治经济稳增长的压舱石，地位不可替代，作用举足轻重。我们必须按照省委、省政府“项目见效年”的部署要求，真正沉下心、扑下身来了解企业生产经营和项目建设的实施情况，继续实施百企百日督促帮扶大会战，对规上企业和应投产项目进行认真梳理，一企一策，组建工作小组，驻企现场指导、帮助、督办困难企业和重点项目，给企业以雪中送炭般的真帮实助。一是降成本。把省、市出台的和即将出台的一系列帮扶企业的政策措施不折不扣地落实到企业和项目上，实实在在为之松绑减负，实实在在地降低企业生产成本，提升产品竞争力。二是占市场。没有市场就没有产品，没有产品就没有生产。对现有企业要组织开展形式多样的促销活动，编制长治工业产品和使用目录，加大宣传推介，四通八达跑营销，见缝插针占市场。要推动市域企业间的联盟合作，互通有无、互惠互补、实现共赢，重点是稳固拓展煤电合作，促使更多的用电大户与电厂建立直供电关系，鼓励企业和政府投资项目在同质同价的前提下优先使用本地产品，加强物流配送的统筹协调，加快网上交易的平台建设，力争全年主要工业产品产销率达到95%左右。三是抓投产。对应建成投产的企业项目要倒排工期，制定任务书、列出时间表，督促企业抓住施工黄金期，完成建设实物量，奠定投产见效的基础。为此，相关部门要变“等着企业来找”为“领着企业去跑”，主动上手、现场办公、专人负责，快节奏地为企业解决报批文件、建设用地、拆迁补偿、环评安评等问题，高效率地为企业组织竣工验收和开展试生产。市政府将设立“项目见效服务办公室”，主要职责是协调解决项目建设中存在的问题，督办项目落地和达效，公开办公电话，公布服务信息，公示项目推进情况，特别是对相关部门和县（市、区）工作不主动、责任不落实、服务不到位、限时不办结的单位和个人点名公开曝光，情节严重的给予党政纪处分。通过一系列有效的帮扶措施，确保85%以上的规上工业企业正常生产；确保年内吉祥煤业60万吨技改、七一新型洗煤、潞宝干熄焦二期等29个传统产业项目和煤焦油加氢、水合肼、无极荧光灯、雅瑞地毯、石油压裂支撑剂、已内酰胺、乙二醇、取向硅钢、低温发电机组、基因工程疫苗等261个新兴产业项目竣工投产、达产达效；确保全年新创办中小微企业1500户，培育“小升规”企业23户、年销售收入超亿元“小巨人”企业15户。

抓企业要素保障。积极为企业提供煤、电、油、气、运等生产条件的基础上，重点解决好资金、土地、人才、创新驱动、园区承载等方面的问题。在解决融资难上，加快构建政府引导、市场主导的政企银对接互助平台，切实解决企业贷款难和“倒贷”难的问题。通过企业联盟以及资产、订单、专利抵押和商标专用权质押等多种方式拓宽融资渠道；推广 “助保贷”、“信义贷”等融资产品，提高金融支持小微企业覆盖率。财政存款与银行贷款挂钩，鼓励银行开发多种金融产品支持各类企业的资金需求；建立财政资金和民间资本融合的担保体系，为企业融资进行担保。在解决用地难上，以县（市、区）为主体大力整合和调剂使用闲置土地资源。今年各县（市、区）闲置土地的调剂使用量和收回量要达到本县（市、区）闲置土地总量的50%以上，以保证新项目用地。同时，强化土地一级管理，只要是好项目并符合土地利用总体规划保证优先供地。在解决人才短缺上，要继续深化与清华大学等著名高校和科研院所的合作，办好长治企业家培训班，着力培养合格的企业管理人才；充分发挥我市职业教育资源优势，不断加强在专业设置、定向培养、师资配备、教学实习等方面与企业的深度合作，加快为企业输送急需的实用人才。在解决创新驱动难上，建立长治技术交易市场，构建高效便捷的技术信息平台。积极为企业“走出去、请进来”的技术合作提供服务、创造条件。引导企业加大科研投入，鼓励企业自主建立技术研发中心和加大专利申请工作。继续发挥好博士（院士）工作站的作用，对中小企业开展的科研活动给予积极扶持。力争全年高新技术企业数量增长30.8%、销售额占规上工业企业销售额的比重达到15.8%，有效发明专利拥有量完成340件，长治高新技术开发区进入国家级行列。在解决园区承载力不足上，关键是要解决园区建设不标准、服务功能不健全的突出问题。按照“九通一平”和“先地下后地上”的原则，加快路、电、气、治污、通讯、物流以及办公研发、文化休闲为主的基础设施建设；建立统一的一站式服务平台，争取工商、税务、环保、住建、人社等部门入园办公。切实发挥园区所具有的资源共享、产业循环、成本节约、土地集约、服务便捷、效益倍增的独特优势，真正把园区办成企业家乐居的创业园、职工安居的生活园、项目争驻的希望园。力争今年建成1个国家新型工业化示范基地和1个省级工业园区，全市工业园区产值达到1200亿元以上，占工业总产值的比重提高2个百分点。

抓企业运行监管。市县政府每月至少召开一次经济运行分析会，加强跟踪调度和分析研判，强化对重点行业、重点企业的运行监管和政策引导，建立监测预警机制，高度关注各项指标任务的完成情况，对经济运行中存在的问题及时有效应对。加强税收征管，开展税收大检查，落实相关部门协助税收责任制，确保应收尽收。加强统计工作，建立统计信息共享机制，完善统计名录库，帮助企业及时办理入库手续，做到应入即入、应统尽统。

抓项目招商引资。我市是典型的投资拉动型经济。项目建设是强化投资最有效的载体，更是推动转型发展最有效的途径。我们必须抓紧抓牢抓实项目建设，决不能松劲。国家和省市鼓励支持上什么样的项目，方向十分清楚，要求非常明确。比如在工业方面重点就是发展现代煤化工、装备制造、节能环保、新能源新材料、电动汽车、新医药和信息产业等战略性新兴产业。在这些方面我们长治有资源、有基础、有条件、有优势，更是“五五战略”中所提出的主攻方向。因此，要积极组织企业参加各类投资贸易博览会，紧紧盯住国内外一流科研院所、高等院校开展产学研对接，紧紧盯住世界500强、国内200强、行业10强、最具成长性的上市公司开展洽谈，重点引进一批科技含量高、带动能力强的项目。每个县（市、区）都要谋划新上2至3个低碳、绿色、环保、真转型的高新技术项目和2至3个高效、生态、真富民的农业产业化项目。我们要切实增强上项目的科学性、前瞻性、主动性，避免盲目性、重复性、依赖性，注重项目的低碳环保性、效益持续性、市场风险性，所以要特别强化项目评估，建立和实施项目联审机制，对新上项目开展前期综合评价，以投入、产出、效益定项目、供土地、给政策。对长期不动工、不建设、不生产的“圈地”项目，必须依规退出，收回土地，不能迁就。

二、大刀阔斧深化转型综改

全面深化改革最现实、最便捷的切入点，就是加快转型综改试验区建设。我们必须严格按照三年实施方案和2014年行动计划，积极开展“转型综改攻坚年”活动，首先从具有全局性、基础性、带动性的几个方面取得改革突破。

在转变政府职能方面，重点是行政审批制度改革。一是要流程再造，为行政审批的繁文缛节“瘦身”。该放的放、该减的减、该并的并、该免的免、该管的真管、该严的真严，明确“权力清单”，严格审批时限，切实体现服务优先、效率优先、规范优先。二是要协调完善，衔接好与行政审批改革相配套的各项工作。相关职能部门要转变观念，及时对内设机构的审批事权进行科学统筹，建立起与行政审批制度改革相适应的工作运行机制；要及时清理本级政府的各类行政性、政策性文件，清除影响行政审批制度改革自我束缚的各种羁绊；要全面清理整顿各类中介机构，加强行业指导和政策引导，规范组织运作，斩断各种“利益链”，真正发挥好“第？健惫焦纳缁岱窆δ堋＝衲耆可笈孪罱胝翊筇迪帧叭小⑷轿弧保⒅鸩酵菩性谙呱笈？月底前全面完成行政审批制度的改革任务。与此同时，今年还要积极做好市县政府机构改革工作，完善政府机构设置和职能配置；完成工商、质监行政管理体制的调整和机构改革。

在土地管理制度改革方面，继续深入推进城乡建设用地增减挂钩、矿业存量土地整合利用和工矿废弃地复垦利用试点，进一步拓展用地空间。加大土地开发整理力度，完成2.8万亩耕地开发任务。强力推进节约集约用地，建立项目用地投入产出效益分析和考核奖励机制，提高单位土地投资强度和产出效益。严格规范土地一级市场，争取实现市本级土地供应的效益最大化。

在金融改革方面，加快金融商务区建设。加快市级转型发展基金的建立和运作。发展各类股权基金。积极推进农信社改制为农村商业银行。鼓励具备条件的民间资本依法发起设立或参股中小型银行。继续推动企业债务融资，鼓励更多符合条件的企业在新三板、天交所等场外交易市场挂牌，扩大市场直接融资规模。大力推进金融生态环境建设，全面提升防范化解金融风险能力。

在财税体制改革方面，改革预算管理制度，将政府收支全面纳入财政预算管理，强化预算约束，推行绩效预算，清理、整合、规范专项转移支付。认真落实煤炭清费立税和资源税从价计征改革。继续推进“营改增”，落实各项税收优惠政策，减轻企业负担。启动实施政府购买公共服务改革试点工作。

在企业改制方面，核心是要解决部分市属国有企业、城镇集体企业和商业企业改制不规范、不彻底、不到位的问题。依照相关政策，新出台全市统一的企业改革实施指导意见，加快对企业的彻底改制步伐，切实解决企业在以往改制过程中遗留至今的各种复杂问题，防止国有资产流失，保障职工合法权益。

三、突出优势发展现代农业

发展现代农业在我市具有明显优势：一是资源条件好，特色产品优；二是农业产业化、农业标准化建设和农产品质量安全工作有基础。我们要扬长避短，围绕重点，加快推进传统农业向现代农业转变。

大力发展特色现代农业。规划建设10个千亩玉米高产示范田。新发展10万亩设施蔬菜、15万亩特色中药材、19万亩干果经济林。新建20个市级现代农业示范园区、30个高标准设施蔬菜示范园区、100个市级“一村一品”示范村，不断提升现代种植业水平。重点推进天一公司肉驴基地、鑫四海种猪基地、潞宝金和生肉鸡屠宰基地、多维牧业种羊基地等9个投资亿元以上的养殖项目，新增100个标准化规模养殖场，扶持培育20个良种繁育基地，不断优化现代养殖业结构。培育壮大金泽生物、太行紫团、沁州黄、林盛果业、方兴农业、沁州绿等列入全市百强企业的27个农业产业化龙头企业，带动全市农产品加工销售收入达到180亿元、增长20%。建成全市农产品质量安全监管与溯源系统，实现对农产品从生产源头到产品上市的全程质量监控。

创新发展现代农业经营体系。倾力创建国家新型职业农民培育整体推进试点市，年内完成1.5万名新型职业农民的培训任务。大力培育专业大户、家庭农场、农民合作社、农业企业等新型农业经营主体，今年培育100个经营规模在500亩以上的种植大户，新建30家省级示范社、50家市级示范社、80家县级示范社，发展300个家庭农场。稳定农户承包权，放活土地经营权，规范县乡村三级土地流转服务体系及县乡两级土地承包经营权纠纷调解仲裁体系建设，支持承包经营权人以经营权向金融机构抵押融资。做好潞城市等农村土地承包经营权确权登记颁证试点工作。深化集体林权制度配套改革，推进森林保险全覆盖。

加大扶贫开发力度。切实做好精准扶贫贫困村和贫困人口的识别工作，重点实施“百企千村”产业扶贫开发工程，以现代农业发展为途径，加快推进武乡油用牡丹种植、沁源好乐草莓、平顺中药材产业开发、壶关旱地西红柿深加工等10个产业扶贫开发项目。扎实做好“千村万人”就业培训工作，对5000名农村贫困劳动力开展就业培训，逐步消灭“零就业家庭”。按照精准扶贫要求，结合农村“五件实事”，继续加强农村困难家庭危房改造，易地搬迁农村贫困人口24472人。扎实开展领导干部包村增收和机关单位定点扶贫工作，实现贫困重点村定点帮扶全覆盖，确保再有4.3万贫困人口稳定脱贫。

致力改善农村人居环境。制定改善农村人居环境规划纲要和2014年行动计划，重点实施好以农村基础设施和公共服务为重点的完善提质工程，以采煤沉陷区治理、易地搬迁、危房改造为重点的农民安居工程，以垃圾污水治理为重点的乡村清洁工程，以美丽乡村建设为重点的宜居示范工程，逐步改善农村生产生活条件。今年重点培育120个市级美丽宜居示范村，建设一批安居乐业的美丽家园。

四、积极稳妥推进城镇化和服务业发展

城镇化的实质是集聚人，服务业的实质是服务人。城镇化建设与服务业发展互为依存、紧密相联。我们必须顺势而为、因势利导，走以人为核心的新型城镇化道路，带动服务业快速发展。要加快城中村、棚户区和重点片区的改造步伐，积极推进城镇户籍制度改革，力争再有6万人进城，全市城镇化率提高1.7个百分点以上。

继续推进主城区功能提升。实施“三河一渠”环城水系综合治理、火车站改造等七大重点工程。新建改造15条市政道路、6座市政桥梁、4座铁路立交桥、6座人行过街天桥、2座互通立交桥、50条背街小巷，完成已开工的3座铁路立交桥、2条快速连接线工程，确保年内“三环八纵十二横”基本贯通。同步规划实施一批停车场、加油（气、电）站、照明、供热、供水、供气、垃圾中转站、公厕、园林绿化等市政基础工程。优先发展公共交通，有效缓解城市拥堵。

继续推进上党城镇群建设。大县城重点围绕加强市政基础设施和公共服务设施建设，做实做强产业园区和核心产业，不断壮大县域经济规模，提高吸纳人口和集聚产业的综合承载能力。重点镇要进一步完善发展规划，因地制宜建设一批各具特色的工业强镇、商贸重镇、文化名镇和旅游大镇，努力成长为县域副中心。中心村要结合美丽乡村建设，完善农村基础设施，积极推进新型农村社区化。每个县（市、区）重点建设1至2个万人以上的示范镇、3至5个3000人以上的示范村。大力推动城市供水、供气、供热、污水和垃圾处理、园林绿化等基础设施向周边县区延伸，坚持建管并举，注重生态宜居，促进城乡一体化发展。

继续推进服务业发展。2013年全市服务业增加值占地区生产总值的比重为30.7%，分别低于全省、全国平均水平9.3、15.4个百分点，在全省排第十位，落后不少但也意味着潜力巨大。发展服务业是我市产业结构优化升级的重要内容，并且大有可为。在现代物流业方面，充分发挥中南、太焦、长邯铁路的交通优势，加快推进长子中南铁路物流港、新易达（二期）等重点项目建设，促进华驰物流项目建成投产；大力发展第三方物流，支持物流企业信息化改造,提升物流业发展水平，促进物流业转型升级。在智慧城市建设方面，依托总投资30亿元的云计算建设项目，整合各部门的信息系统和业务应用，建立信息共享与业务协作机制，打造综合信息集成平台，建成标准规范、入口统一、功能完备、方便快捷、安全可靠的“一站式”服务平台，提供全方位的智慧政务、智慧民生、智慧产业服务。在商贸流通领域方面，加快建设区域商业中心，发展大型综合类仓储式商场、购物中心，重点打造城隍庙古玩藏品、昌盛电子产品、云步餐饮等特色商贸街区；大力发展连锁经营、物流配送、电子商务、网上购物等现代流通新业态，重点培育一批骨干流通企业，全面提升消费水平。同时，加快推进中介服务、创意设计、管理咨询等新兴生产性服务业发展。在文化旅游业方面，利用“太行情、上党行”的央视宣传广告，形式多样地塑造长治的旅游品牌，继续做好壶关“冲关大峡谷”、武乡《太行山》实景剧等宣传推介活动，重点推进太行八路军文化产业园、太行山大峡谷、太行红山、仙堂山、通天峡、灵空山、北莱沟滑雪场、荫城古镇等景区景点建设；做好平顺太行水乡、神龙湾、红色西沟景区资源整合工作；加快炎帝综合体、长治游客集散中心建设，全面提升旅游业发展水平。

五、毫不松懈狠抓安全生产管理和生态环境治理

安全工作和生态环境工作都是人命关天的大事要事。这两件事是一切工作的基础，一切发展的前提，丝毫马虎不得，丝毫放松不得。

全力抓好安全生产。强化政府监管责任，就是要全面贯彻2013年7月18日习近平总书记在中央政治局第28次常委会上，就安全生产工作做出的重要指示：各级党委和政府要增强责任意识，落实安全生产负责制。要落实行业主管部门直接监管，安全监管部门综合监管，地方政府属地监管。坚持管行业必须管安全，管业务必须管安全，管生产经营必须管安全，而且党政同责，一岗双责，齐抓共管。这是党中央向全党、全社会提出的关于安全工作的总要求。按照这个总要求，在市委的领导下，今年年初市政府出台了关于2014年安委会成员单位的工作职责及任务分解意见，进一步完善和明确了49个部门的194项工作职责，形成了抓安全工作必须全行业覆盖、履安全责任必须全领域覆盖、保安全生产必须全方位覆盖的工作格局。每个行业、每个部门、每个领域都要履好职、尽好责，进一步强化安全工作人人重视、安全生产人人有份、安全稳定人人尽力的责任意识，真正把政府监管责任落到实处，保长治久安。落实企业主体责任，关键是企业的各项安全管理制度和安全生产责任是否持之以恒、不折不扣地抓到位。重点是企业的安全生产例会是否定期召开，专职安全生产负责人是否配齐配强，安全隐患是否定期排查整改，安全设施是否完备，生产一线是否每天都有领导带班，安检队伍是否健全，每个职工对自己的安全生产操作是否熟知熟会等，这些都是企业必须做到和不断加强的，也是政府监管必须经常检查和严格督查的。总之一句话，无论是政府的监管责任，还是企业的主体责任，落实责任重于泰山。

强力抓好大气污染防治和节能减排。坚决打好“向污染宣战、享碧水蓝天”十大环保攻坚战，对规划区内重污染企业必须关、停、并、转；燃煤锅炉必须更新改造，油气、黄标车、扬尘、餐饮油烟、垃圾、秸秆焚烧等必须加大治理；逐步完善城市垃圾无害化处理；建立重污染天气监测预警体系，加强应急处置和联合执法，严厉打击超标排放、超量排放、渗坑排放、恶意排污等违法行为，确保主城区PM 10 、PM 2.5 浓度分别比上年下降2%和4%。在煤炭、焦化、冶金、电力、化工、建材等行业大力实施先进技术的节能改造；严格限制高耗能产业发展，淘汰落后产能150万吨；推进一批工业固废利用重点项目的达产达效，提高利用率2个百分点。推广节能产品、倡导低碳生活、鼓励绿色出行，确保年度节能减排的各项目标任务全面完成。

着力改善水环境质量。继续推进浊漳河流域生态环境综合治理，开工建设长治市人工湿地工程，严厉查处、取缔浊漳河沿岸废水排放不达标企业，分阶段完成化工企业生产用岩溶水置换地表水工作，严格地表水跨界断面水质目标考核办法，确保国省控断面水质稳定达标。推进山西大水网骨干项目辛安泉供水改扩建工程建设；加强辛安泉水源地保护，年内完成浊漳河南源文王山地垒河段防污整治工程。建成主城区应急备用水源主体工程。加快推进市污水处理厂二期工程建设，确保今年下半年投入使用。提升县区城镇污水处理能力。加强农村饮用水质量检测，保障农村饮水安全。

大力实施造林绿化工程。突出抓好“八大”林业生态工程建设，完成营造林40万亩，确保森林覆盖率再提高1个百分点，森林蓄积量增加100万立方米。加大园林绿化建设力度，配套跟进市政道路、单位、小区的整体绿化。建立生态文明建设目标评价考核体系，完善生态补偿机制，全面推进采空区、沉陷区、水土流失区、煤矸石山的生态环境治理修复，严格控制主城区周边的煤炭开采，促进全市生态环境持续改善。

六、扎扎实实做好民生和社会事业工作

民生和社会事业人人共享、关系千家万户。为此我们不仅要尽心尽力，更要尽职尽责，不遗余力地推动各项民生和社会事业的不断进步，努力适应人民群众的新需求。

优化就业创业环境。实施更加积极的就业政策，加强人力资源市场建设，形成覆盖城乡的公共就业服务网络。拓宽高校毕业生就业渠道，开展好实名登记、专场招聘、春风行动等就业援助活动。统筹抓好农村劳动力、城镇就业困难人员和退役军人的就业工作。出台创业型城市建设实施意见，建立创业基金，落实税费减免、社保补贴、创业指导等扶持政策，鼓励创业就业，每个县（市、区）至少建立一个创业孵化基地。

均衡发展教育事业。整合教育资源，通过组建长治职业教育学院和幼儿师范高等专科学校，推进职教园区建设。基本普及高中阶段教育。实施义务教育学校标准化建设，加快农村义务教育薄弱学校改造。为全市3万名农村义务教育阶段寄宿制学生提供高标准免费营养餐。探索以名校组建教育集团、联盟校等办学模式，让更多的学生享受优质教育资源。全市义务教育阶段学校校长、教师交流实现全覆盖，交流比例达到10%，城区通过义务教育发展基本均衡评估认定，沁源、屯留、襄垣和郊区通过省级义务教育标准化认定。新建、改扩建公办幼儿园18所，改造农村幼儿园35所，完成主城区3所公办幼儿园建设。

加强医药卫生事业。健全全民医保体系，完善重特大疾病医疗保险和救助制度，全面推开新农合大病保险工作。巩固和发展县级公立医院改革成果，稳步推进市级公立医院改革，积极推动基本药物制度向非政府办基层医疗卫生机构延伸。加强全科医生转岗培训。深化“平安医院”创建。启动建设长治市精神卫生中心。做好人口计生工作，巩固发展“好娃娃”工程成果，全面开展出生缺陷干预救助健苗工程，提高出生人口素质。

健全社会保障体系。强化社会保险扩面征缴工作，整合城乡居民基本养老保险制度，积极推进“五险”统征。企业退休人员基本养老金再提高10%。城镇居民医保和新农合财政补助标准再提高40元，达到320元。城乡居民最低生活保障水平每人每月分别提高25元、22元。健全社会救助体系，增强社会救助兜底功能，为建国前参加革命工作的老工人发放医疗补助，为领取失业金的失业人员发放取暖补贴，为重度残疾人发放护理补贴和生活补贴。稳定物价水平，完善社会救助和保障标准与物价上涨挂钩的联动机制。

加快保障房建设。完善基本住房保障和供应体系，今年再开工建设城镇保障性住房12400套、基本建成11100套，完成投资35亿元。规范保障性住房管理、分配，推进公共租赁住房和廉租住房制度并轨运行。加大普通商品住房供给，促进房地产市场健康发展。

繁荣发展文化事业。继续实施文化惠民工程，深入开展各类公益文化活动，大力推进市博物馆、科技馆、图书馆、文化艺术中心等文化基础设施建设，进一步巩固提升公共文化服务体系示范区创建水平。实施六府塔、城隍庙、上党门、观音堂四大古建修复工程和各类古村落的保护。加强非物质文化遗产的保护和传承。鼓励文化创新，打造文化精品。创优五大文化院团发展条件。举办好第八届全国“牡丹奖”曲艺大赛、长治微电影国际大赛和上党神话故事艺术节。启动市体校搬迁工作。推动对市体育中心以及相关体育资源的市场运营工作。

加强和创新社会治理。认真实施 “六五”普法，不断深化“法治长治”建设。扎实推进社会“网格化”管理，畅通社情民意诉求渠道，做好人民调解和社区矫正工作，有效预防和化解社会矛盾。尽快落实“一村（社区）一警”联系走访活动，完善社会治安防控和公共安全体系，依法严密防范和惩治各类违法犯罪活动。加强应急管理和防灾减灾能力建设，妥善应对各类突发事件。依法管理民族宗教事务，促进社会和谐稳定。开展无欠薪市、县创建活动，加大工资支付监控和欠薪查处打击力度，确保农民工工资按时足额发放。严格食品药品安全监管，加强基础设施建设，建立统一的全程追溯体系，确保人民群众饮食、用药安全。今年要做好国家园林城市、卫生城市、文明城市的复审检查工作。

进一步加强气象、地震、档案、保密、国防教育、人民防空、民兵预备役、双拥共建、外事侨务、对台、地方志等工作，充分发挥工会、共青团、妇联等人民团体的桥梁纽带作用，关心支持妇女儿童、残疾人、老龄、红十字会、慈善救助等事业。

各位代表，为民务实清廉是政府自身建设的核心内容，也是检验政府自身建设水平高低的重要标准。加强政府自身建设，一是要扎扎实实开展好党的群众路线教育实践活动。坚持问题导向,抓住关键、找准要害、深入剖析、建章立制，着力解决关系群众切身利益和联系服务群众“最后一公里”问题。每个政府工作人员都要通过教育实践活动祛“四风”之害，补精神之“钙”，切实解决门难进、脸难看、话难听、事难办的问题。深入学习纪兰精神、段爱平先进事迹，真正把为民务实清廉的价值追求深深根植于思想和行动之中，尽心竭力履职尽责，勤勤恳恳为人民服务，做出让人民满意的一流业绩。二是要驰而不息推进法治政府建设。自觉接受人大及其常委会的监督，主动接受政协的民主监督，提高办理人大代表议案、建议和政协委员提案的质量，广泛听取民主党派、工商联、无党派人士和人民团体意见。支持法院、检察院依法独立公正行使职权。严格按照法定权限和程序行使权力、履行职责，推进政务和办事公开。坚持科学民主决策和权力制约协调机制，强化行政执法责任和过错追究，完善行政复议制度，构建决策科学、执行坚决、监督有力的权力运行体系，推进法治政府建设再上新台阶。三是要毫不放松打造高效廉洁政府。深入落实中央“八项规定”和国务院“约法三章”，厉行勤俭节约，反对铺张浪费。牢固树立过紧日子的意识，花好财政的每一分钱。规范公务接待，“三公”经费支出再压缩10%。加强行政监察和审计监督，深入开展民主评议和政风行风电视问政。加强效能督查和述职述廉工作。全面落实党风廉政建设责任制，严格执行廉洁从政的各项规定，始终保持惩治腐败的高压态势，努力实现干部清正、政府清廉、政治清明。

各位代表，上党从来天下脊，老区辉煌定有期。让我们在省委、省政府和市委的坚强领导下，紧紧依靠全市人民，坚定信心、凝聚共识，真抓实干、锐意进取，为实施“五五战略”、加快转型跨越、率先全面建成小康社会而努力奋斗！
